# Robotic winding of wet-spun fibers for biofabrication of aligned, perfusable, muscle-based tissue

**DOI:** 10.1088/1758-5090/aeb3b9

**Published:** 2026-11-02

**Authors:** Joseph R Broomhead, Kyle Y Kunisaki, Johanna M Teegarden, Kallie L Frett, Caleb D Vogt, Aidan S Johnson, Siddhant Yelwande, Elizabeth C Yao, Zahra Khosrowpour, Michael Kyba, Angela Panoskaltsis-Mortari

**Affiliations:** 1 Department of Pediatrics, Blood and Marrow Transplant and Cell Therapy Division, University of Minnesota—Twin Cities, Minneapolis, MN 55455, United States of America; 2 Department of Pediatrics, Blood and Marrow Transplant and Cell Therapy Division, MSTP, University of Minnesota—Twin Cities, Minneapolis, MN 55455, United States of America; 3 Department of Pediatrics, Blood and Marrow Transplant and Cell Therapy Division, MN Robotics Institute, University of Minnesota—Twin Cities, Minneapolis, MN 55455, United States of America; 4 Department of Pediatrics, Blood and Marrow Transplant and Cell Therapy Division, Department of Medicine, MN Robotics Institute, University of Minnesota—Twin Cities, Minneapolis, MN 55455, United States of America

**Keywords:** muscle tissue engineering, wet-spinning, Robotics, ventricle, gastroesophageal junction

## Abstract

Structural alignment of muscle and integrated vasculature enables the function of geometrically complex tissues in the body, such as the gastroesophageal junction and the ventricles. However, replicating this complexity is an ongoing challenge. Described here is the wet-spun & robotically articulated placement (WRAP) method for deposition of aligned cellular fibers on scaffolds, resulting in large structures fabricated from the inside-out. WRAP enables complex, multi-oriented muscle and vascular placement by creating a continuous cellular fiber through wet spinning, and then using a robotic arm programmed with a set of tissue profiles to maneuver the fiber around an organ scaffold. Wet-spun fibers, consisting of muscle cells combined with endothelial cell fibers via double-core spinning, exhibited the ability to mature, align, contract, and initiate tubulogenesis, creating perfusable fibers. Further, the robotic arm could accurately create physiologically relevant fiber patterns with high precision. The potential to generate perfusable blood vessels alongside functional muscle is paramount to fabricating muscle-based constructs. The WRAP method offers a high throughput and versatile solution to the scale-up problem plaguing the biofabrication of muscle-based tissues.

## 1. Introduction

Creating tissue that accurately replicates normal healthy tissue for implantation is one of the main goals of tissue engineering. Fundamentally, the combination of cells, scaffolds, and incorporated signaling molecules are thought to be the building blocks of a successfully engineered tissue construct. However, as the field has matured, increased effort has focused on the importance of the microstructure and form of the tissue, particularly in terms of alignment. Cells and extracellular matrix materials, such as collagen, are aligned within various organs to accomplish the role of the tissue [1, 2]. This is most apparent in the musculoskeletal tissues, where these alignments are best arranged to either generate or withstand large forces [3–6]. Other tissues experience specialized forces, including the cardiovascular system where generation and withstanding of blood pressure and flow is important, and the gastrointestinal system which must likewise act in a coordinated manner to produce the forces necessary for mechanical digestion and propulsion of food [7, 8]. Thus, there is increased interest in developing methods of tissue engineering which can allow tight control over the alignment of individual cells or groups of cells within a tissue.

Several methods have been developed to accomplish these broad goals. One of the most popular methods utilizes 3D bioprinting. The most common 3D bioprinting approach uses a pneumatic, extrusion-based technology that deposits cells in specific orientations to achieve alignment [9]. While highly effective for creating single aligned layers, 3D bioprinting remains limited in its ability to create multiple aligned tissue layers, which is necessary for organ production. Other commonly used approaches include patterned substrates and external cues such as mechanical stimulation or electrical fields [10–12]. While these methods are effective to promote cellular alignment, they also struggle to create complex multilayered patterns. Furthermore, many of these methods encounter significant barriers to the scale-up necessary for organ production [13–16]. Bioprinting requires excessive amounts of time and resources, patterned substrates are difficult to fabricate at the size needed for organs such as the heart, and external cues are typically limited in ability to adequately reach all areas of a large construct, be they biochemical or biomechanical. In addition to the structural complexity, large engineered tissues require vascularization to properly mature and remain viable. However, incorporation of vascular networks remains a challenge for many of these strategies [17].

Because of the general limitations of these methods, when trying to create larger more complex tissues with overlapping areas of complex alignment, additional methods have been investigated. One general approach to build upon the successes of single cell patterning is to create small tissues, which are then assembled into more complex structures in a second manufacturing step [18]. In this manner, simple, repeatable building blocks can be produced using well-understood processes. These building blocks have been investigated at a variety of scales, ranging from microtissues and organoids with only 10–100 s of cells to larger macroscale tissue sheets. One increasingly common strategy utilizes pre-fabricated microtissues for 3D bioprinting applications. Specifically, microtissues are incorporated into a bioink and 3D printed into complex patterns [19–21]. However, this only creates a single layer of alignment, and the resulting tissues often consist of discontinuous microstructures rather than fully integrated constructs. Another common approach utilizes tissue sheets which can then be manually stacked, folded, or shaped into complex structures to recreate physiological alignment [20, 22, 23]. While effective, this method again encounters significant scale-up issues. It requires a large amount of manual labor and automation of these processes remains a challenge, which increases the risk for inconsistencies in the resulting structure. More recently, woven tissues made from biofabricated tissue fibers or constructs have emerged as a promising strategy [24–27]. Although effective in creating mechanically strong tissues, woven tissues inherently interlace the fibers resulting in an isotropic alignment throughout the construct. Furthermore, all of these methods fail to incorporate a controllable, perfusable vascular network, multilayer tissue alignment, and automation, which is a critical bottleneck as the size and complexity of these constructs increases.

Here, we present the wet-spun & robotically articulated placement (WRAP) method, a platform designed to address key bottlenecks in organ engineering (figure 1). Using the microfluidic system we developed in a previous study, we generated functional, continuously aligned fibers of smooth muscle, two skeletal muscle subtypes, and perfusable vascular tissues using endothelial cell fibers [28]. We further demonstrated that a six-degree-of-freedom (6 DOF) robotic arm can precisely position these fibers to recreate physiologically relevant alignment patterns on organ scaffolds. WRAP enables the efficient and reproducible fabrication of multilayered muscle tissues while incorporating the vascular architecture necessary to support physiologically relevant tissue dimensions. Considering the prealigned microtissues (PAM) described in previous work, this method enables longer morphological structures like myoblasts and blood vessels and overall improves scalability [28]. By expanding the reach of established approaches for unidirectional cellular alignment with the generation of complex, overlapping alignment patterns, WRAP provides a versatile strategy for engineering structurally and functionally sophisticated tissues.

**Figure 1.**
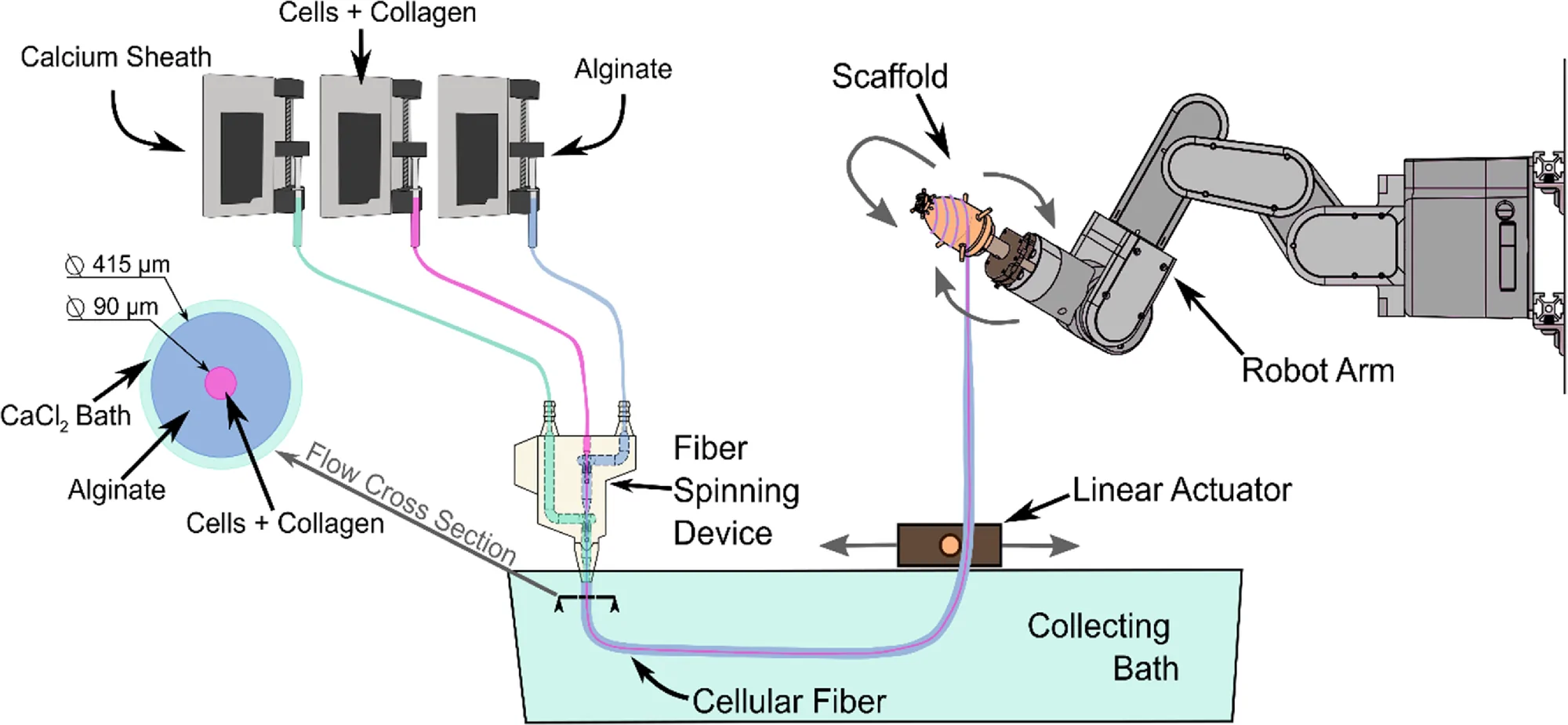
WRAP biofabrication method. Diagram detailing the fabrication steps, structure of the biological fibers, and use of the six-degrees-of-freedom robotic arm and linear actuator to precisely place fibers on the construct scaffolds. Individual components not to scale.

## 2. Methods

### 2.1. Cell culture

C2C12 murine myoblast cells (ATCC, CRL-1722), passage 2–10, were cultured in a proliferation media formulation on tissue culture plastic at 37 °C and 5% CO_2_. Proliferation media consisted of high-glucose Dulbecco’s modified Eagle’s medium (DMEM) (Gibco, 11 965–092) supplemented by 10% FBS (Genesee Scientific, 25–514) and 100 *µ*g ml^−1^ Primocin (Invivogen, ant-pm-2). Post fiber spinning, cells went through a proliferation phase for 3 d, using the media formulation mentioned above, and on day 3 differentiation medium was introduced. Differentiation medium consisted of high-glucose DMEM (Gibco, 11 965–092) supplemented with 10% Horse Serum (Thermo Fisher, 16 050 122), and 100 *µ*g ml^−1^ Primocin (Invivogen, ant-pm-2).

Primary Human Esophageal Smooth Muscle cells (HESMCs) (ScienCell, 2710) were cultured in a proliferation media formulation on tissue culture plastic at 37 °C and 5% CO_2_. Proliferation media consisted of smooth muscle cell medium (ScienCell, 1101) supplemented with 2% FBS (ScienCell, 0010), 1% Smooth Muscle Cell Growth supplement (ScienCell, 1152), and 1% penicillin/streptomycin solution (ScienCell, 0503). Post fiber spinning a differentiation medium was introduced. Differentiation medium consisted of high-glucose DMEM supplemented with 10% FBS (Genesee, Scientific), and 100 *µ*g ml^−1^ Primocin. iPS-derived human skeletal myoblasts (HSkM) were isolated using the procedure outlined in supplementary methods 1, which included culture of human iPSCs, teratoma generation in mice, tissue dissociation, teratoma harvest, isolation of myogenic progenitor cells through fluorescence-activated cell sorting, expansion and cryopreservation of myogenic progenitors, and *in-vitro* myogenic differentiation and characterization. This is a modified procedure as seen previously [29]. The cells were cultured in a proliferation media formulation on tissue culture plastic coated with 0.1% gelatin at 37 °C and 5% CO_2_. Proliferation media consisted of Skeletal Muscle Cell Basal Medium (SkBM-2) (Lonza Biosciences, CC-3246) supplemented by the SkGM-2 SingleQuots supplements (Lonza Biosciences, CC-3244). Post fiber spinning, cells went through a proliferation phase for 3 d, using the media formulation mentioned above, and on day 3 differentiation medium was introduced. Differentiation medium consisted of high-glucose DMEM (Gibco), supplemented with 2% FBS (Genesee Scientific), 1% Glutamax (Thermo Fisher, 35 050 061), 1% ITS-A (Thermo Fisher, 51 300 044), 1% penicillin-streptomycin (Sigma-Aldrich, P4333) [29].

Primary human umbilical vein endothelial cells (HUVECs) (ScienCell, 8000) were cultured in a proliferation media formulation on tissue culture plastic coated with 0.1% gelatin at 37 °C and 5% CO_2_. Proliferation media consisted of endothelial cell medium (ScienCell, 1001), supplemented with 5% FBS (ScienCell, 0025), 1% of Endothelial Cell Growth supplement (ScienCell, 1052), and 1% of penicillin/streptomycin solution (ScienCell, 0503). Post fiber spinning, a differentiation medium was introduced. Differentiation medium consisted of the proliferation media supplemented with 10 ng ml^−1^ of vascular endothelial growth factor [30] (R&D Systems, BT-VEGF).

### 2.2. Immunofluorescence microscopy

Single fiber samples were fixed for 30 min with 4% paraformaldehyde (PFA) at room temperature, washed 3X for 5 min with DPBS and used immediately or stored at 4 °C for later analysis. Samples were first permeabilized in 0.125% Triton for 30 min. Blocking was performed for 1 h with a blocking buffer of 10% Horse Serum (Thermo Fisher Scientific, 16050122) and 1% bovine serum albumin (BSA) (Sigma-Aldrich, A3294). Primary antibodies were diluted in the blocking buffer and incubated overnight. HESMC samples were incubated with Anti-SM-MHC, anti-Calponin-1, anti-CX43. C2C12 and HSkM samples were incubated with anti-MF-20, anti-*α*-sarcomeric actinin, anti-laminin. HUVEC samples were incubated with anti-Von-Willebrand–factor, anti-CD31, and anti-VE-cadherin. All samples included an additional Ki-67 stain. Fibers were washed 3X for 5 min with DPBS before adding secondary antibodies diluted in 1% BSA and incubated at room temperature for 1 h. Secondary antibodies used were goat-anti-mouse Cy3 and goat-anti-rabbit DyLight 488. Fibers were then washed 3X for 5 min with DPBS. All fibers were incubated in DAPI for 10 min, and subsequently washed 3x for 5 min with DPBS. Some fibers were incubated in iFluor™ 488-conjugated phalloidin at 1:1000 dilution without other antibodies to evaluate cellular alignment. Stained fibers were then rinsed 3X for 5 min in DPBS before mounting with mounting media (Vector Laboratories, H-1900-10). Summary of the antibodies and dilutions are included in supplementary table 1. All samples were then imaged using a Nikon A1R Multiphoton Confocal Microscope (figures S1–S3).

Cell alignment studies were performed following the protocol as previously described [28]. Five images were taken on the Nikon A1R Multiphoton Confocal Microscope for each of HESMC (day 7), HSkM (day 14), and C2C12 (day 14) fibers labeled with f-actin and DAPI. For each sample, a randomly generated region of interest (ROI) of 100 × 100 pixels was generated along the fiber to analyze f-actin. The OrientationJ plugin, from FIJI (NIH), was used with the distribution setting, cubic spline fit, and a sigma value of 2. For nuclear alignment, the DAPI-labeled fibers were imported into NIS Elements and a custom script was used to segment the nuclei in 3D. The software returned the coordinates, spherical equivalent diameter, major, minor, and minor 2 axes, and major axis orientation in *x*–*y*. The segmented objects under 3 *µ*m in equivalent diameter were filtered out. A 2nd order polynomial fit was performed on each fiber to create an equation used to define a 200 × 200 pixel randomized ROI along the fiber. Nuclear orientation probability was assessed by fitting nuclear orientation angles into 5 degree bins and plotting the probability of each angle relative to the fiber orientation in GraphPad Prism.

Dual fiber samples were fixed for 30 min with 4% PFA at room temperature, then washed 3X for 5 min with DPBS and used immediately or stored at 4 °C for later analysis. Gelatin Methacryloyl (GelMA) was synthesized following existing procedures [31] using Methacrylic anhydride (Sigma-Aldrich, 276 685), and Gelatin (Millipore Sigma, G1980) with dialysis tubing (Spectrum Laboratories, 142 252). The fiber samples were then embedded in 10% GelMA and crosslinked. Samples were permeabilized in 0.125% Triton for 30 min and then blocking was performed for 1 h with 10% Horse Serum, 1% BSA. Primary antibodies (anti-MF-20 and anti-Von-Willebrand-factor) were diluted in blocking buffer and incubated for 24 h. The embedded fibers were washed 3X for 5 min with DPBS before adding the secondary antibody (goat-anti-mouse Cy3, dilution 1:500) diluted in 1% BSA and incubated at room temperature overnight. The embedded fibers were then washed 3X for 5 min with DPBS. All embedded fibers were incubated in DAPI for 20 min, and subsequently washed 3x for 5 min with DPBS. Some embedded fibers were incubated in iFluor™ 488-conjugated phalloidin without other antibodies. The samples were then immersed in a glycerol and imaged using a Nikon A1R Multiphoton Confocal Microscope.

Post-alginate lyase morphology was assessed through immunofluorescent staining. For human skeletal muscle, a tissue construct was prepared on a custom spool used for contraction testing. For HESMC, fibers were wound around a gastroesophageal junction (GEJ) frame and cultured in a vented glass culture vessel (PhytoTechnology Laboratories, C11892). After 6 d of incubation, alginate lyase (8 *µ*g ml^−1^ concentration) in DPBS was added to the culture spools. After 1 h, the media was replaced with fresh media and the cells were cultured for an additional 8 d. For the skeletal muscle spool, it was first embedded in 12% GelMA under UV light to preserve its alignment, then was fixed with 4% PFA, then was stained with phalloidin-iFluor™ 647 Conjugate (Cayman Chemical Company, 20555) at a 1:1000 dilution and DAPI and immersed in glycerol under the same procedure outlined for the dual-fiber samples. The construct was imaged on the Nikon A1R Multiphoton Confocal Microscope. The HESMC fibers around the GEJ were encapsulated with GelMA and UV crosslinking was then applied to the entire construct to hold the fibers in place. The construct was removed from the scaffold and immersed in 4% PFA for 45 min. The construct was cut to allow for sections of interest to be imaged on a microscope slide, and the construct was stained with phalloidin-iFluor™ 488 Conjugate. The construct was imaged using a Leica DMI3000-B.

### 2.3. Gene expression

Quantification of the expression of genes related to tissue maturity was performed using TaqMan assays (Applied Biosystems). First, the alginate shell was dissolved from the fibers using the following protocol: alginate lyase at a 4 mg ml^−1^ concentration was diluted to 1:50 in culture media and incubated for 1 h at 37 °C in the incubator. The samples were then spun down and aspirated to isolate the tissue pellet. The mass of the tissues was measured, and an appropriate volume of TRIzol reagent (Thermo Scientific, 15596018) was added. The fibers were homogenized using a rotor-stator homogenizer, and the solution was stored at −80 °C. RNA extraction was performed with chloroform according to the TRIzol manual, and the RNA was collected and washed using RNeasy (Qiagen) spin columns cDNA was produced using a SuperScript III kit (Invitrogen, 18080044) and a thermocycler. The qPCR plates were loaded with cDNA samples and the following TaqMan assays for each cell type. For HESMC, the target genes were *MYH11, CNN1*, and *GJA1*. For HSkM, the target genes were *MYH1, LAMA1*, and *ACTA4*. For C2C12, the target genes were *MYH4, LAMA1*, and *ACTA1*. For HUVECs, the target genes were *VWF, CD31*, and *CDH5*. Three reference genes were used for every cell type which were *B2M, GAPDH*, and *HPRT* for accurate normalization following methods outlined in the literature [32, 33]. Finally, the quantitative polymerase chain reaction (qPCR) was performed using a LightCycler 96 (Roche). Analysis was performed using the ddCt method, where the three reference genes were used as the reference and freshly wet-spun fibers were used as the reference samples for each cell type. Summary of the probes used are included in supplementary table 2. One way ANOVA and Tukey HSD as a post-hoc analysis were performed as statistical tests to determine significance between days for each gene and tissue type.

### 2.4. Myotube and sarcomere maturation

To quantify nuclear fusion and myotube length for both day 14 C2C12 and HSkM fibers, Imaris (Oxford Instruments) was used to create a surface of the DAPI stain for nuclei count and a surface of the MF20 stain for myotubes (figure S4). For nuclear fusion, the Imaris statistical tool ‘shortest distance to surface’ was used to compute the distances between each segmented nucleus and the closest myotube surface. For myotube length, the Imaris statistical tool ‘BoundingBoxOO Length C’ was used to calculate the length of the longest principal axis on each myotube. The data was exported and analyzed with Excel (Microsoft). Lastly, sarcomere lengths were calculated using ImageJ by analyzing areas of fluorescent images containing distinct sarcomeres (figure S5). Specifically, lines were drawn through the sarcomeres and pixel intensity along these lines were used to calculate the lengths.

### 2.5. Robot winding

The wet spinning setup was modified from previous work to include a sterile 2 l coagulation bath (Cambro, 22LPCW135) which would allow for the full range of motion of the robot [28]. Fibers were spun into the bath, and the spinneret position was moved periodically to keep the fibers from tangling during the robot winding process.

A Mecharm 270pi robot (Elephant Robotics) was modified by adding a 7th axis stepper motor (Nema, 11HS04-0504S) which allowed for continuous rotation of the end effector. Code was written in Python to control the robot servos, the 7th axis, and a linear actuator attached to the robot.

Scaffolds were prepared in Solidworks® following the normal human anatomy of the target features. The scaffolds were designed to have removable sections to easily extract the fiber construct from the scaffold after incubation. The scaffolds were built to mimic geometrically complex structures in the body and to prove the automated robot winding method could assist in the scale-up of engineering large organ constructs.

Scaffolds were sliced in Preform slicing software and then 3D printed using an SLA printer. Surgical Guide Resin (Formlabs), an autoclavable and biocompatible resin, was used to construct the scaffolds on a Formlabs Form 4B SLA printer.

The 3D-Cross scaffold was built to have removable arms in order to slip the construct off the main axis. This construct was built to showcase the complex winding ability of the system. The winding relied on the fiber being strong enough to hold its own slack, and by utilizing gravity, the fiber would hang vertically from the construct. The robot was programmed to spin the 3D cross scaffold as the robot held the construct at varying angles, thus wrapping the scaffold tightly with the fiber. A 3D cross construct was produced using C2C12 fibers, under the 17 d procedure outlined above. On day 13 of differentiation, the fibers were exposed to 4 mg ml^−1^ Alginate Lyase for one hour to leave only the C2C12 muscle fibers. On day 14 post-differentiation, 20% GelMA in DPBS, 0.25% LAP was added to the construct and crosslinked using a 395 nm light to lock the fibers together. The construct was then removed from the scaffold and fixed in 4% PFA for imaging, with the GelMA Lock holding the fibers in place. An acellular construct was also created for ct imaging, modified by being submersed for 18 h in 2.5% Iodine Potassium Iodide (Carolina Biological supply, 872 795), 7.5 mM CaCl_2_, 100 mM NaCl in DI H_2_O. The I_2_KI is traditionally used in taxonomic imaging of organs by enabling more soft-tissue contrast under x-ray.

The GEJ construct was built to replicate the three main layers of aligned smooth muscle that could be found in this anatomy. Gastric clasp and sling fibers were represented by utilizing pegs on the scaffold to hold the fibers in a geometrical position when spun onto the scaffold. By using the continuous 7th axis of the robot, an approximation of circumferential esophageal fibers was made through mandrel winding. The construct was designed with a removable section that would allow for the fiber-laden construct to be removed from the scaffold and rounded edges to prevent the fibers from being cut during incubation. Human esophageal smooth muscle was wound onto the construct, starting with the clasp fibers, then the sling fibers, then the circumferential fibers. The construct was incubated for 6 d, then Alginate Lyase (Sigma-Aldrich, A1603) was applied to dissolve the alginate shell. GelMA and crosslinking was then applied to the construct to hold the fibers in place. The construct was removed from the scaffold and fixed in 4% PFA for macro-imaging. An acellular construct was produced, then submerged in I_2_KI for 18 h for ct imaging. Seven acellular GEJ constructs were created independently using only the robot arm. The GEJ constructs were placed on a scaffold a set distance away from a phone camera. The clasp and sling winds were analyzed between constructs using ImageJ measurement tools to measure three angles on the side of the construct.

The heart ventricle scaffold was designed with a removable cap, four pegs for the fiber to hold its structure with, and a shape mimicking the human left ventricle. The heart’s anatomy is made up of multiple layers of aligned cardiomyocyte fiber. With the progression into the wall of the ventricle, the helical angle of these fibers changes by approximately 180°, which ultimately results in a ‘wringing’ effect of the heart to better pump blood [34]. Following dtMRI images of the heart’s anatomy, the ventricle scaffold was designed to replicate helical windings of −30°, 0°, and 30° progressively through the thickness of the ventricle wall. The robot was programmed to deposit the fiber along these helical paths using a linear actuator to fine-tune the helical structure through the position of the draping fiber. After each helical wind, the robot flipped the construct, with the fiber catching on the posts on the scaffold, then the 7th axis rotated in the opposite direction to repeat the same helical angle to complete the −30° winds. For circumferential windings, the scaffold was treated as a mandrel and fiber was wound around the scaffold. Then the +30° winds were completed on top of the circumferential fibers in a similar fashion to the −30° winds to complete the ventricle model.

### 2.6. Packing density and overlap

Cross sectional images of acellular fibers spooled around small spools were taken on an EVOS M700 Imaging System and analyzed using Python coding software (figure S6). The edges of the fibers were outlined manually to avoid background noise from crossing fiber interference. The manually processed images were then analyzed in Python to determine the total area of the fibers and the total area that the fibers occupy. The packing density was calculated using the equation:${\mathrm{packing}}\,{\mathrm{density}} = \frac{{{\mathrm{fiber}}\,{\mathrm{area}}}}{{{\mathrm{total}}\,{\mathrm{area}}}}.$

The ratio of the alginate shell radius to the collagen core radius was determined using ImageJ to analyze cross sectional images taken on an EVOS M700 Imaging System. A theoretical number of layers required for confluence in the final construct assuming strictly parallel deposition of the fibers is calculable. Given that p is the packing density of the fibers, *D* is the diameter of the alginate shell and d is the diameter of the core fiber, the number of layers required (*L*) is given by the following:$L = \frac{D}{d} \div p.$

C2C12 and HSKM fibers were spun around the spools used for contraction with 100 and 200 winds. The fibers were incubated until day 13 post-differentiation, then were treated with 8 *µ*g ml^−1^ Alginate Lyase to dissolve the alginate shell. On day 14, the fibers around the spool were transferred to forceps while preserving the construct’s ring structure. The ring construct on the forceps was then dipped in a bath of sterile 12% GelMA, 0.1% LAP in DMEM. The construct was UV Crosslinked for 1 min to embed the ring construct in the GelMA hydrogel. The forceps were removed from the embedded ring construct, and the construct was removed from the bath and placed into a 6 well plate. The construct was fixed in 4% PFA for 45 min, then washed with DPBS three times. The resulting embedded ring constructs were then cut cross-sectionally with a surgical blade creating thin sections that were imaged on the EVOS M700 Imaging System.

### 2.7. Micro-CT scanning

The robot-spun samples prepared with 2.5% I_2_KI were aspirated and rinsed with DPBS. Each construct was scanned in open air in the micro-CT Machine (H225 X-teck XT, Nikon). The *µ*CT scans were reconstructed, reconstructed, and analyzed using VG Studio.

### 2.8. Acellular tensile testing

Acellular fibers were spun using the previously described method [28]. The sample was prepared by isolating a single fiber on the spool and cutting. To perform the tensile tests a custom silicone chamber was designed and the ends of the fiber were wound around the peg of this silicone chamber to create a loop (figure S7). To secure the fiber around the peg a 50% w/v PEGDA hydrogel with 0.5% w/v LAP (Lithium phenyl-2,4,6-trimethylbenzoylphosphinate) was pipetted into the chamber and cured with UV for 30 s. 4 ml of 10 mM CaCl_2_ was added to the silicone chamber. The silicone chamber with the prepared fiber sample was then placed on the Mach-1 mechanical testing machine (Biomomentum). A custom hook grip was screwed into the force gauge, and was inserted into the loop of the fiber sample and the sample was preloaded to 0.005 ± 0.004 gf. The Mach-1 settings for each experimental run was a max amplitude of 50 mm and a velocity of 0.5 mm s^−1^. A video was recorded of each experimental run and the failure mode and peak force was recorded. A total of 12 samples were measured.

### 2.9. Contraction testing

Contraction experiments were performed on the skeletal muscle cell lines (C2C12 and HSkM), which were wet-spun onto custom spools designed to create continuous tissue rings. Cells were cultured according to the methods described in this study and spun as previously described in [28]. Then, using the robot system each spool was wound for 100 full revolutions around the spool. These were cultured for 14 d in 6-well plates, as previously described, however on day 13 the alginate was dissolved. The alginate dissolution protocol was as follows: alginate lyase, at a 4 mg ml^−1^ concentration, was diluted to 1:50 in culture media to dissolve the alginate shell over the course of 1 h at 37 °C in the incubator. The dissolved alginate + culture media solutions were then replaced with fresh media.

To perform the contraction experiments the DMT Organ Bath System was used to measure contraction force of the tissue, and the 820MO & Grass S88X Dual Output Square Pulse Stimulator was used to stimulate the tissue. First, the Organ Bath System was calibrated using a bridge and T-balance. For each tissue type, a sample was used to determine the optimal stimulation parameters. Prior to contraction testing, samples were incubated in Krebs solution for 15 min at 37 °C (120 mM NaCl, 4.7 mM KCl, 1.2 mM MgCl_2_-6H_2_O, 1.2 mM NaH_2_PO_4_-H_2_O, 25 mM NaHCO_3_, 2.5 mM CaCl_2_, 11 mM D-Glucose, 2 mM EGTA in DIH_2_O). Immediately after, samples were placed onto the contraction forks in the organ bath system, which was filled with a 40 mM KCl solution at 37 °C. The contraction forks were initially separated by 10 mm, were then increased to a distance of 15 mm as a uniform preload across samples. When transferred to the forks, the ring constructs from the 100 winds resulted in a ring construct roughly 4 fibers thick. When collecting data, markers were placed in the LabChart software which were synchronized with the start and end of each electrical stimulus. The LabVIEW software had a sampling rate of 1000 Hz. The HSkM samples were stimulated at a frequency of 20 Hz with 10 V. The C2C12 samples were stimulated at a frequency of 25 Hz at 10 V. Stimulation ran for approximately 30 s, and the tissues were allowed to relax for 120 s. The first contraction trial was omitted from data due to the fibers reorganizing on the forks during, and after, the first stimulation and the recorded force skewing as a result. For the HSkM fiber rings, 21 independent samples were analyzed with 9 contraction events each. For the C2C12 fiber rings, 11 independent samples were analyzed, each with 9 stimulations analyzed to produce the contraction waveform. Each independent sample’s mass was recorded to normalize data between samples of different sizes. The samples were analyzed using a MATLAB script which smoothed the data, averaged each waveform, normalized each independent sample to estimate the force of a single fiber, then produced a final contraction-relaxation curve for each cell type.

### 2.10. Perfusion

HUVECs were cultured until confluent, then mixed into 5 mg ml^−1^ Collagen at a ratio of 60 million cells ml^−1^. Fibers were spun using the method described previously [28]. Samples were taken on days 0, 3, 7, and 14 for immunofluorescent staining. The day 14 fibers formed a tubular vessel, and were placed in a petri dish lid containing DPBS for perfusion on an EVOS M700 Imaging System. The petri dish lid was short enough to allow for a shallow 15° needle approach angle into the sample. A nanomanipulator (Discovery Technology International) was modified with a 3D Printed mount intended to hold a needle at 30°. A 10 *µ*m glass capillary tube (Fivephoton Biochemicals, MGM 1 A-5) was glued using cyanoacrylate (Loctite® 4011™) to a 2 mm diameter silicone tube attached to a precision dispenser tip (Nordson, 7018 178). The glass capillary tube construct was placed into a clamp on the nanomanipulator (figures S8(A)–(C)). The endothelial cell fibers were prepared by cutting them into 1–2 cm fragments using a Barraquer scissor (Weck, 2320) under the microscope. The ends of the endothelial cell fibers were observed to assess whether they were open or collapsed. 10 ml of human blood (400 billion cells ml^−1^) was drawn into a tube (Becton Dickinson, 367874) with 158 USP Units of sodium heparin. Sample blood was mixed and then drawn into a 3 ml syringe, which was attached to the dispensing tip and the glass capillary tube was primed with blood. Using the nanomanipulator, the glass capillary needle tip was then inserted into the lumen of the endothelial cell fiber. The microscope was positioned to take images (figure S8(D)) and videos (videos S1 and S2) during perfusion. The syringe was pushed to dispense blood through the endothelial cell fiber.

## 3. Results

### 3.1. Fiber alignment

Primary HESMCs, mouse skeletal muscle cells (C2C12), and iPSC-derived HSkM were cultured and wet-spun onto collection spools for incubation similar to our previously described method [28]. To assess the ability of the wet-spinning technique to induce muscle alignment, f-actin and nuclei were stained to determine the probability of alignment to the fiber axis (figures 1(A)–(C)).

All images used for f-actin alignment were z-stack projections. The f-actin alignment in the skeletal muscle fibers was analyzed on day 14, and in HESMC fibers on day 7. The f-actin stains for each of the muscle subtypes (figures 2(D)–(F)) show aligned filaments in the cytoskeletal actin structure along the fiber’s long axis for each cell type. The nuclei of each cell type (figures 2(G)–(I)) demonstrated a high degree of alignment as shown in their corresponding histograms. Although the HESMC fibers resulted in lower cytoskeletal alignment than the C2C12 and HSkM cells, it is clear from figure 2(C) that the filaments are tightly packed, and the orientation of the nuclei (figure 2(I)) demonstrate cellular alignment along the fiber’s long axis. Thus, these results demonstrate that the fabrication platform can align various muscle cell types in a single direction, enabling them to be controllable by the orientation of the fiber.

**Figure 2.**
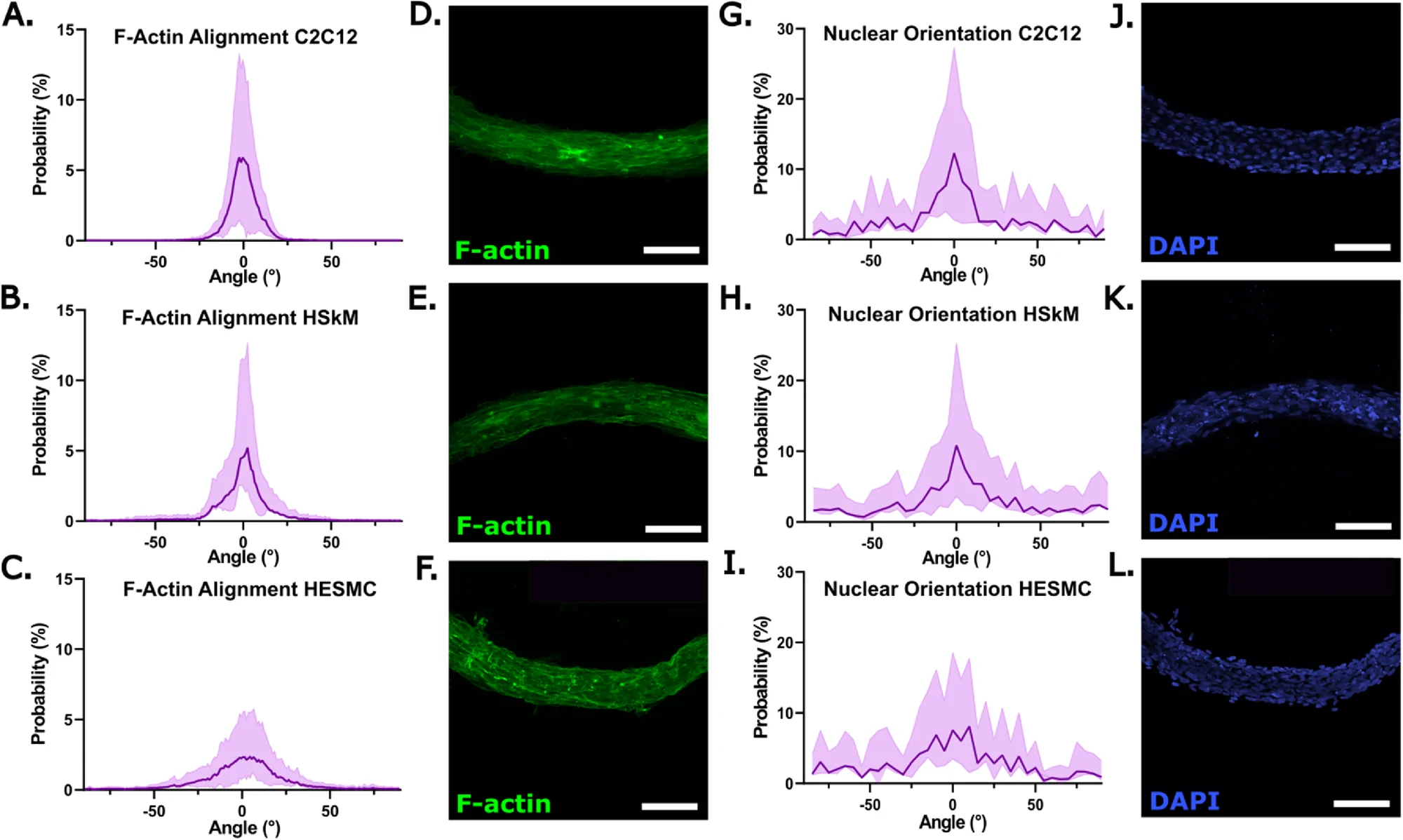
Wet-spinning generates aligned muscle fibers. (A)–(C) histogram of the alignment data for the day 14 C2C12 fibers, day 14 HSkM fibers, and day 7 HESMC fibers (*n* = 5). (D)–(F), representative f-actin-stained fibers used for image analysis for alignment data; scale bar, 100 *µ*m. (G)–(I) histogram of the nuclear alignment data for the day 14 C2C12 fibers, day 14 HSkM fibers, and day 7 HESMC fibers, (*n* = 5). (D)–(F), representative DAPI-stained fibers used for image analysis for alignment data; scale bar, 100 *µ*m.

### 3.2. Muscle function

HSkM and C2C12 fibers were morphologically and functionally analyzed for their maturity. Day 14 fibers were stained for *α*-sarcomeric actinin, a marker for sarcomeres, and for MYH11, a marker for myotube formation. Sarcomeres are the essential structure for contraction in skeletal muscle. Myotube structure was analyzed utilizing a custom image segmentation pipeline developed in the Imaris software (figure S4). Specifically, myotube length indicates maturity, myotube thickness is an indicator of contractile strength, and fusion index is a measure of differentiation [35, 36].

On day 14, both the human skeletal muscle fibers and the C2C12 muscle fibers exhibited sarcomere expression, indicating that the tissues were sufficiently matured for contraction experiments (figures 3(A) and (B)). More sarcomere images are included in supplementary figure 5. In the C2C12 samples, myotube length, myotube width, and sarcomeric spacing, were consistent with values reported in the literature [37–39] (figure 3(C)). Similarly, the fusion index aligned with values reported in literature [37, 40] (figure 3(C)). For the human skeletal muscle samples, the myotube thickness, fusion index, myotube length, and sarcomere length were slightly lower than the values expected from the literature [39, 41–43] (figure 3(C)).

**Figure 3.**
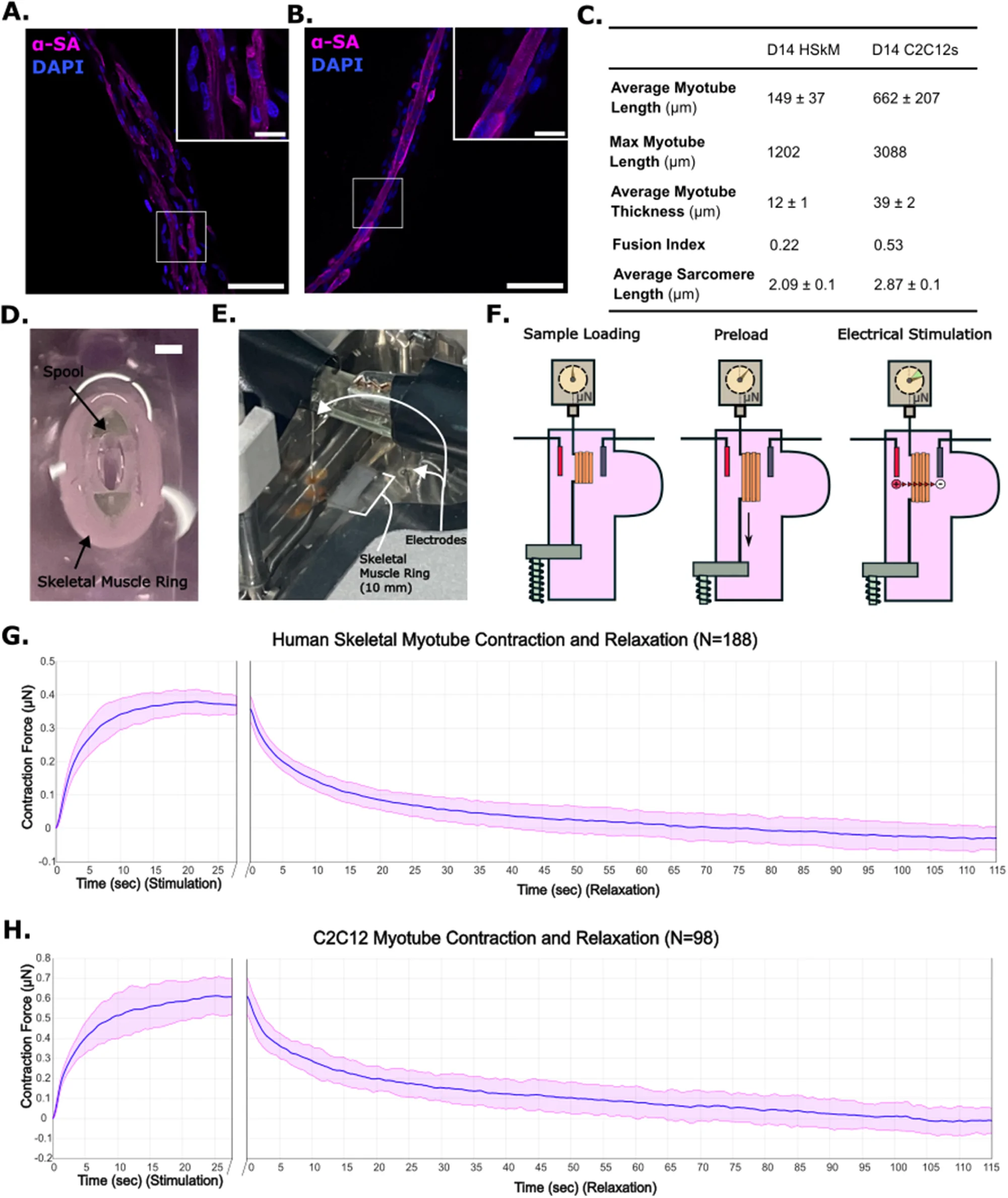
Wet-spun skeletal muscle cells develop into functional muscle tissue. (A) day 14 immunofluorescence image of a human skeletal muscle fiber with higher magnification of the sarcomeres in the top right corner. Top right scale bar, 25 *µ*m. Bottom right scale bar, 100 *µ*m. (B), day 14 fluorescent image of a C2C12 muscle fiber with higher magnification of the sarcomeres in the top right corner. Top right scale bar, 25 *µ*m. Bottom right scale bar, 100 *µ*m. (C) table containing values for morphological characteristics of the fibers with myotube analysis as measured from a large tiled scan of fibers stained for DAPI and MF-20 and sarcomere analysis from a series of close-up images of *α*-sarcomeric actinin and F-actin stained fibers. (D) image of wet-spun skeletal muscle fibers wound around a contraction spool with the alginate shell removed. (E) image of the contraction experiment with the stimulation electrodes and tissue in the organ bath system. (F) illustration of the contraction experiment methods. (G) and (H) compiled contraction graphs for human skeletal and C2C12 samples. The dark blue line is the mean value and the light pink shading is the standard deviation from all samples. Time break indicates when the electrode stimulation was stopped.

For contraction testing, wet-spun fibers were wound onto a custom contraction spool (figure 3(D)), and matured to day 14. Twenty-four hours prior to contraction experiments, the alginate shell was dissolved, allowing for a ring of muscle to form (figure 3(D)), which was then loaded into the contraction testing apparatus (figures 3(E) and (F)). Tissue was then stimulated electrically to induce a tetanic contraction (figures 3(E) and (F)).

The contraction data for both the human skeletal and the C2C12 samples demonstrated the expected tetanic profile for force versus time [44–46]. The human skeletal muscle fibers exhibited lower maximal contraction force per fiber, which is consistent with the weaker sarcomere expression and thinner myotubes, both of which are indicators of force generation in skeletal muscle. Nonetheless, these results demonstrate the system’s ability to generate functional and mature skeletal muscle fibers. Furthermore, these results are consistent with the fluorescent images (figures S2 and S3) that demonstrate the lower myotube expression and sarcomere patterning, and more loosely packed myotube morphology seen in the human skeletal muscle samples on day 14. Unprocessed data can be seen in figure S9. The qPCR data also demonstrated that the tissues were expressing genes associated with maturation for each tissue type (figure S10).

### 3.3. Robot function

The robot winding process was developed to enable placement of fibers in a controlled and versatile manner. To establish the wrapping movements and foundational operating parameters for efficient implementation, three different scaffolds were used: 3D cross, GEJ, and a ventricle. These structures would test the system’s ability to create complex patterns around scaffolds of various shapes. In addition, key parameters were investigated, including the number of alginate-shelled fiber layers to produce a single muscle tissue layer and the maximum height the robot can manipulate the fiber as it comes out of a coagulation bath.

Figures 4(A)–(H) illustrate the ability of the WRAP method to create complex movements around the organ scaffold, and lay down fibers in complex, physiologically relevant patterns such as circumferential alignment, clasp and sling fiber deposition, and angled helical alignment. Additionally, the packing density analysis demonstrated that 5 layers of alginate-shelled fibers result in 1 layer of packed cellular core fibers once the alginate sheet is removed (figure 4(I)). Non-composite sodium alginate fibers produced in the wet-spinning method have previously been reported as weak [47, 48], so to determine the maximum range of the robot, the reachable fiber height from the bath surface needed to be measured. 10 tensile tests were performed while recording the ultimate tensile strength (UTS). Utilizing the lowest UTS value, the maximum height was found to be 53 cm, which acted as a bounding box for the robot’s movements (figure 4(J)). Lastly, to determine the ability of the robot system to reproducibly lay down fibers at specific orientations, the angles of the fibers wound onto a ventricle and GEJ scaffold were examined. For the ventricle, the robot was programmed to deposit the expected angles of −30° and +30°, which was in alignment with the results in figure 4(K). For the GEJ, the fibers were laid down at angles with less than 5 degrees of variation between independent scaffolds (figure 4(L)). Thus, these results demonstrate the successful development of a robot system to precisely, and reproducibly, lay down fibers.

**Figure 4.**
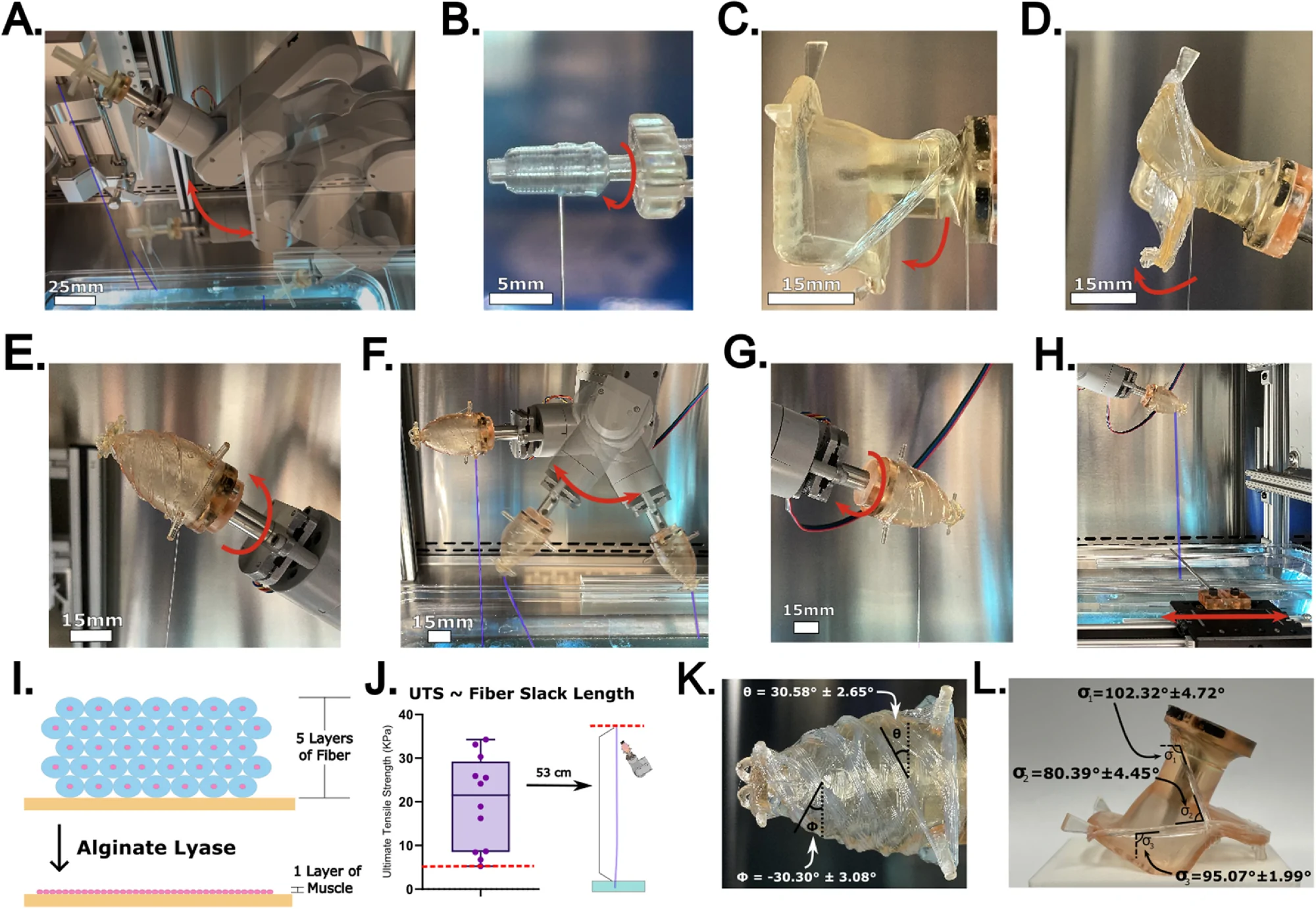
Precision wrapping of fibers using a 6 DOF robotic arm. (A) demonstration of the robot system’s freedom of movement. (B) robot performing circumferential winding around a spool. (C) demonstration of the robot system laying down sling fibers around the GEJ scaffold. (D) demonstration of the robot transitioning between sling and clasp winds around the GEJ scaffold. (E) demonstration of the robot laying down fibers in a helical pattern at +30° around the ventricle scaffold. (F) demonstration of the robot transitioning to lay down helical fibers at −30° onto the ventricle scaffold. (G) demonstration of the robot system laying down fibers at −30° onto the ventricle scaffold. (H) demonstration of the linear actuator system’s movement and its coordination with the movement for precise winding patterns. (I) schematic to depict the packing density results which revealed 5 layers of alginate fibers created 1 full cellular core layer. (J) box and whisker plot of the tensile testing data to determine the maximum height an alginate fiber can reach out of the bath. Lowest ultimate tensile strength was used, denoted by the red dotted line, and showed the fiber can reach a height of 53 cm before breakage. The max height the robot system reaches in the system is 50 cm when performing ventricle winds. (K) angle measurements of the winding patterns on the ventricle scaffold with standard deviation (*n* = 15). (L) angle measurements of the winding patterns on the GEJ scaffold. With standard deviation (*n* = 7).

With the robot movements and parameters established, the fiber winding patterns were verified using *µ*CT imaging. The 3D cross demonstrated the system’s ability to create complex winding patterns around the *X, Y*, and *Z* axes. As seen in figure 5(B) C2C12 fibers were cultured onto the scaffold to verify the system’s compatibility with cell culture. In figure 5(C), it is clear that fibers were successfully wrapped around all three axes. The GEJ demonstrated the system’s ability to recreate complex physiological fiber patterns, which is a current challenge for organ engineering [8]. This was highlighted by figures 5(E) and (F), where the sling, clasp, and circumferential winds are easily seen with cultured HESMC fibers and a *µ*CT scan. Lastly, the heart ventricle was used to examine the system’s ability to create complex helical windings, which has been a long-term challenge in the field of cardiac engineering [36, 49]. WRAP could replicate the transmural fiber alignment throughout the myocardium, as demonstrated by winding fiber layers at helical angles of −30°, 0°, and +30° around the construct. This was highlighted by the *µ*CT image shown in figure 5(I), where all angles are all clearly shown via the enlarged section, and quantified in figure 4(K). These images demonstrate, in detail, the high precision of the robot system to create continuous complex patterns.

**Figure 5.**
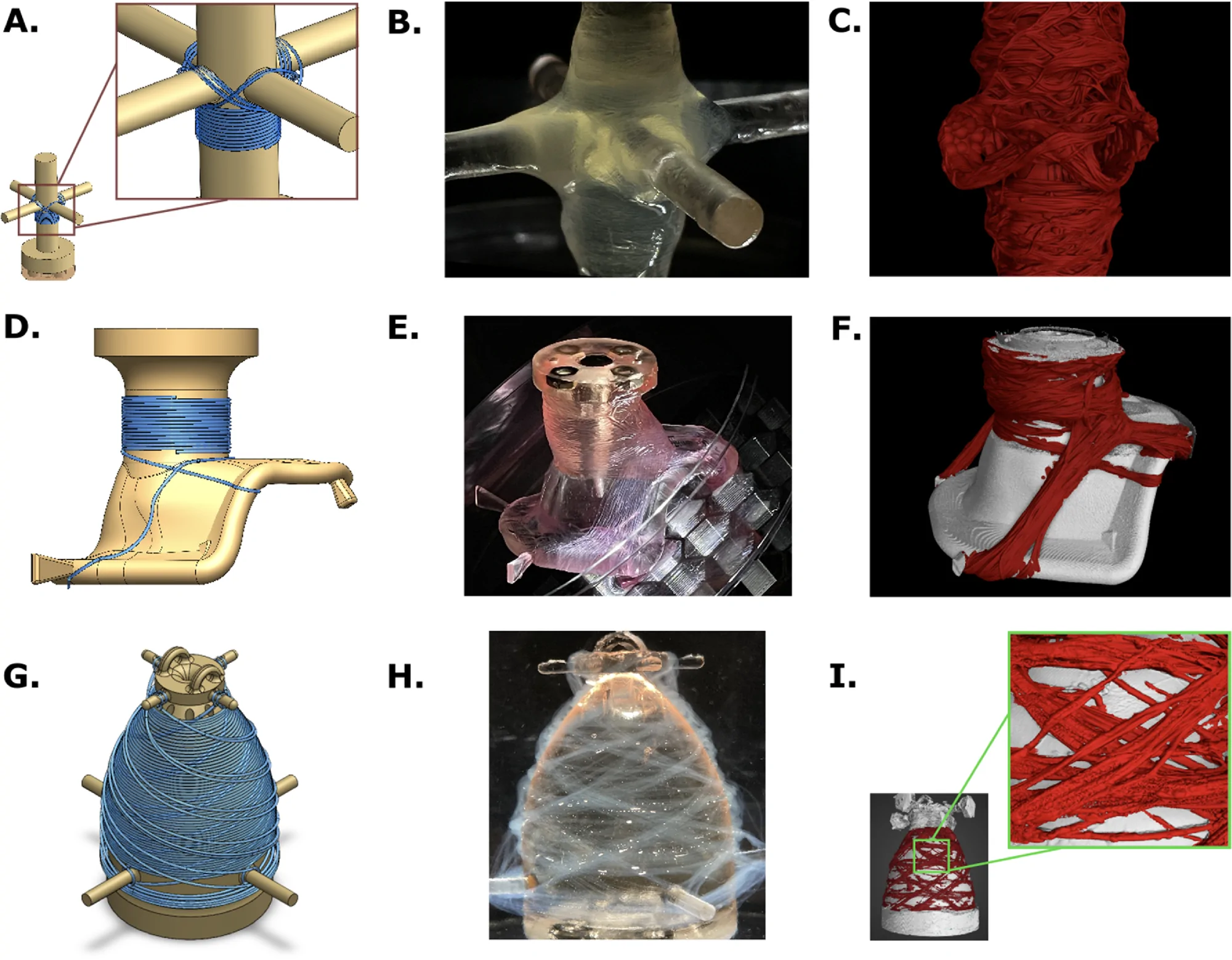
Demonstrations of wrapped fibers on designed constructs. (A) SolidWorks® model of 3D cross with fiber winding (blue) on the scaffold (47 × 47 × 52 mm). (B) 3D cross wound with alginate fibers loaded with a collagen core and C2C12 cells incubated for 17 d. (C) *µ*CT image of the 3D cross with collagen-core alginate fibers showing the complex winding pattern created by the robotic system. (D) SolidWorks® model of the GEJ with representative alginate fiber in blue to demonstrate the winding pattern (47 × 40 × 37 mm). (E) GEJ with HESMC fibers wrapped around the construct and incubated for 7 d. (F) *µ*CT image of the GEJ construct wound with collagen-core alginate fibers to represent the physiologically accurate winding pattern created by the robotic system. (G) SolidWorks® model of a heart ventricle with a representative wrapped alginate fiber in blue (53 × 53 × 51 mm). (H) ventricle wrapped with a collagen loaded alginate fiber. (I) *µ*CT image of the ventricle wrapped with a collagen-core alginate fiber to represent the physiological winding pattern from −30° to +30°.

Additionally, the behavior of tissue fibers on the organ frames following alginate dissolution was examined (figure S11). Overlapping tissue fibers retained their directionality, while fibers wound in the same direction exhibited partial fusion.

### 3.4. Vascularization and dual-spinneret

To enable the WRAP system to generate viable thick muscle tissues, strategies for vascularization were investigated. HUVECs were integrated into the wet-spinning process and cultured for 14 d, after which immunofluorescence staining and perfusion studies were performed to assess vascular conduit formation and functionality. Building on these findings, a dual-core spinneret was developed to incorporate a vascular component adjacent to the muscle tissue fibers. The resulting constructs were evaluated through imaging and immunofluorescence staining for tissue maturation markers.

On day 14 of maturation, the HUVEC fibers were stained for CD31 and DAPI to image their 3D structure. It is clear from figures 6(A) and (B), and supplemental figure S12, tubulogenesis occurred between day 0 and day 14 resulting in the formation of a continuous lumen. The lumen diameter was approximately 40 *µ*m, approximating the size of arterioles, which regulate flow into the capillary beds [50]. Through adjustments to the core flow shown in our previous work, this method also demonstrated the ability to create vessel diameters down to 10 *µ*m, similar to the size of capillaries, which is a current area of interest within the field [28, 51].

**Figure 6.**
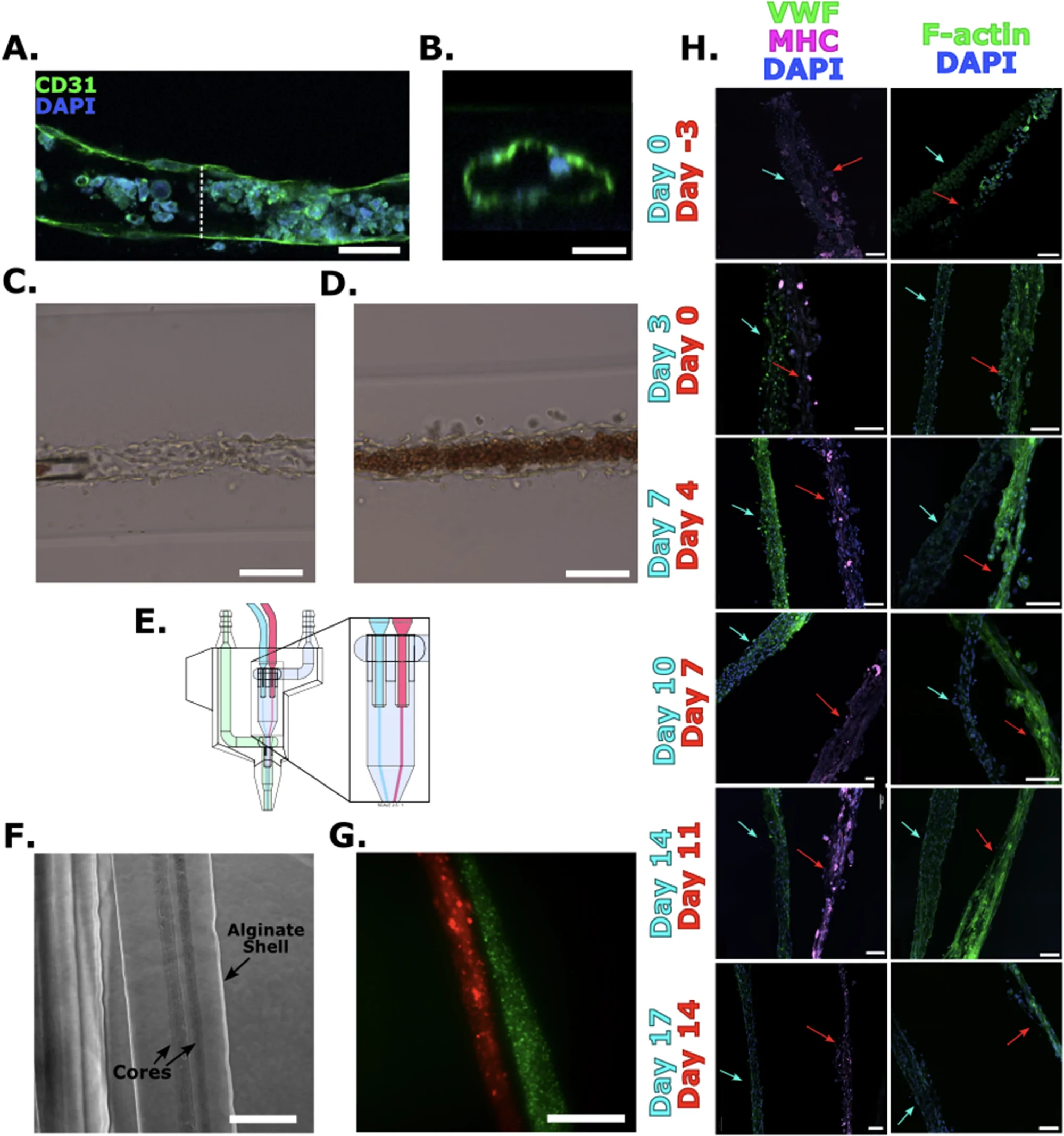
Vascularized fibers can be created using a dual-spinneret. (A) Top-down image of a day 14 HUVEC fiber; scale bar, 50 *µ*m. (B) Long-axis view of the HUVEC fiber. The dotted line in (A) represents the location from which the fiber in image (B) was reconstructed; scale bar, 20 *µ*m. (C) Image of HUVEC fiber used for perfusion study before human blood was perfused; needle containing blood is on the left hand side of the image; scale bar, 50 *µ*m. (D) Image of the HUVEC fiber after blood was perfused through; scale bar, 50 *µ*m. (E) SolidWorks® drawing of the dual spinneret design with the flow of the cores highlighted by the zoomed box. (F) Phase-contrast image of the multi-core fiber with the outer alginate shell and the two cores indicated by the arrows; scale bar, 500 *µ*m. (G) Fluorescence image showing the dual core fibers loaded with red and green fluorescent beads; scale bar, 500 *µ*m. (H) Stitched max intensity immunofluorescence images of the dual core fibers loaded with HUVEC and human skeletal muscle cells. Blue (left) arrows indicate the HUVEC fiber and red (right) arrows indicate the human skeletal muscle fiber; scale bar, 100 *µ*m.

To determine the structural integrity and perfusability of these fibers, a glass microneedle filled with human red blood cells was inserted into one end of a day 14 fiber. Figures 6(C) and (D) demonstrates a fiber before and after perfusion, while supplementary videos 1 and 2 provide videos of this process. The video revealed the Fåhræus–Lindqvist effect within the flow profile, consistent with physiological microvascular bolus blood flow [52]. Additionally, many of the fibers demonstrated a continuous lumen, one of the largest being 4 mm in length. Thus, proving that these fabrication methods could create perfusable, structurally sound, vessels approximating the flows seen within the body.

To attempt to incorporate these capillary structures into other tissues a dual core spinneret was created (figure 6(E)). This would allow for the addition of a vascular core alongside the muscle tissue that can be established within this system [28]. To validate the design, fluorescent red and green microbeads were added to the two cores, individually, and then imaged under brightfield (figure 6(F)), and fluorescence (figure 6(G)). These images clearly demonstrate two separate uniform cores within the alginate fiber, establishing that the design functioned as expected.

Next, HUVEC and human skeletal muscle cells were added to the two cores, respectively, and imaged over the course of maturation (figure 6(H)). The images demonstrate the ability of the system to align and mature both tissues. From the initial day of seeding to the final day of maturation, the cells become tightly aligned with increased expression of maturation markers. The non-parallel patterns seen in figure 6(H) between the tissues is due to the twisting and large three-dimensional nature of the dual core fiber.

## 4. Discussion

A variety of tissues in the body rely on highly organized cellular alignment for physiological function. Importantly, these architectures are rarely aligned as a single layer and their angle often differs in three dimensions across the tissue thickness. These patterns consist of multiple continuously aligned and vascularized tissue layers which work together as building blocks to form a macro structure. For example, the myocardium exhibits a unique transmural change in myocardial fiber orientation that is nearly 180°, which contributes to efficient cardiac contraction and maximal ejection fraction [36, 49, 53]. Similarly, the GEJ consists of multiple tissue layers of aligned smooth muscle that are essential for proper function and the maintenance of the high pressure barrier between the esophagus and stomach [8]. To date, recreating these complex patterns remains a challenge in tissue engineering. While many groups have successfully created aligned constructs, and even engineered a beating ventricle, these methods are often limited to single layers or a few stacked layers [52, 54, 55]. Although these methods are promising, they face many challenges to the scale-up necessary for whole organs. In addition, these methods, including our original method, have difficulty incorporating multiple aligned tissue layers due to fabrication time, manual labor, and vascularization challenges [28].

The WRAP method presented here demonstrates the ability of this system to overcome many of the current challenges with organ engineering and is the next-generation technology of the original PAM-based printing approach [28]. First, the fiber fabrication method demonstrated its ability to align and mature various muscle cell types, creating a single continuous fiber with long myotubes not inhibited by the length of the construct, something other methods lack [21]. Furthermore, while the qPCR data demonstrated minimal statistically significant differences in expression across the days for each tissue type, it is clear that the genes associated with maturation were expressed. However, the HSkM fibers exhibited reduced myotube characteristics than expected. This can likely be attributed to the fact that iPSC-derived myogenic progenitors exhibit immature phenotypes and require longer maturation times to reach functional and structural maturity [56–59]. Additionally, it was shown the fiber spinning method can facilitate vascularization and induce tubulogenesis, as evidenced by the perfusion results and the physiological-like blood flow seen within the fiber’s lumen during perfusion. The adjustments of fiber core diameter, as seen in previous work, alongside the variation in arteriole diameter observed in this system, demonstrate the system’s future application toward creating blood vessels of different sizes [28]. This was taken a step further with the dual-core spinneret, an advancement from our original single core spinneret, which allowed for the coculturing of this vascular fiber component alongside muscle inside the same wet-spun fiber [28]. Thus, the system has the ability to overcome the vascularization issue and allow for the creation of large multilayered organs seen in the body. Lastly, the spinning system facilitates scalable manufacturing.

Pairing these results with the winding capabilities of the robot arm demonstrates this system’s potential for whole-organ fabrication. The winding studies highlight the robot’s ability to create highly complex fiber patterns with multiple layers onto the scaffolds. Importantly, the robot recreated the physiologically relevant architectures of two of the most structurally complex muscle-based organs in the body, the GEJ and the heart ventricle. Furthermore, these patterns can be reliably reproduced across multiple layers, with the potential to easily control size and layer number by modifying the robot code and increasing material input. Because the fabrication of tissue is automated, each structure is easily reproducible, allowing for efficiency and consistency unmatched by manual labor. This capability is further enhanced by the incorporation of vascularized, perfusable fibers to the muscle tissue, enabling the creation of larger more complex engineered tissues. In addition, the continuous nature of the fiber fabrication process allows for the creation of uninterrupted tissue structures rather than discrete tissue segments that many methods use, including our original PAM-based method employed [28]. Furthermore, the automation allows for efficient customization of alignment patterns, organ geometry, and tissue architecture. Finally, preliminary studies demonstrating that skeletal and smooth muscle fibers retain their directionality following alginate dissolution, even when wound in non-parallel patterns, further support the system’s ability to generate and maintain complex tissue architectures.

Overall, the WRAP method addresses many key challenges in tissue engineering, provides a foundation for whole organ engineering, and is the next step from our original work [28]. It solves the issue of discontinuous tissue segments, lack of vascular tissue, fabrication time, and single-layered geometrical simplicity that our previous method faced [28]. However, this study has limitations. First, the organ frames exist as a showcase of the robot technology, thus cells were not incorporated into the fiber winding onto the ventricle scaffolds and no large organs were functional. As a result, the structural integrity and behavior of the tissues on the organ frames during long-term culture, remain unknown. Second, the current winding process for the 3 cm diameter ventricle takes upwards of 60 min limiting the scale up potential and potentially the viability of cardiomyocytes. While this method enables scale-up geometrically, there is still a limitation brought on by the large quantity of cells needed to create a human-sized organ; as the contraction spools of 100 winds only had around 400 *µ*m of muscle fiber. Lastly, minimal studies, aside from short term perfusability, were conducted on the vascular tissue. More work such as barrier function, long term perfusability of oxygen carried by blood cells, and the creation of branching networks needs to be conducted. Also, the interactions of the vasculature and muscle tissue were not examined and future studies will determine if the vasculature is capable of delivering oxygen and nutrients to the muscle tissue. The WRAP method, as presented here, is an engineering demonstration due to the lack of functional studies of the tissues on the organ frames. Further development is required to determine its ability to create fully functional whole organs, but it provides a foundation for creating vascularized, geometrically complex tissues.

## 5. Conclusion

The work presented here demonstrates a novel platform for the scale-up of geometrically complex and muscle-based tissue engineering. Aligned and functional wet-spun fibers of various muscle cell types can be precisely deposited along the surface of a scaffold to build an organ layer-by-layer. Complex patterns of macro-scale muscle alignment, found in organs such as the GEJ and the ventricles of the heart, can be recapitulated through the WRAP method. Additionally, perfusable blood vessels can be integrated into the structures as a component of dual core fibers, thus enabling the scale-up of tissue thickness and size. Future studies should focus on the mechanical integrity, long-term tissue maturation, and functional performance of the whole construct, before and after alginate dissolution. Further robot optimization will help address limitations of the technology and will push the field closer to the ultimate goal of engineering large, vascularized, structurally complex tissues.

## Data Availability

All data that support the findings of this study are included within the article (and any supplementary files). Supplementary data 1 available at https://doi.org/10.1088/1758-5090/aeb3b9/data1. Supplementary data 2 available at https://doi.org/10.1088/1758-5090/aeb3b9/data2. Supplementary data 3 available at https://doi.org/10.1088/1758-5090/aeb3b9/data3.
